# ESCAPS study protocol: a feasibility randomised controlled trial of ‘Early electrical stimulation to the wrist extensors and wrist flexors to prevent the post-stroke complications of pain and contractures in the paretic arm’

**DOI:** 10.1136/bmjopen-2015-010079

**Published:** 2016-01-04

**Authors:** Joanna C Fletcher-Smith, Dawn-Marie Walker, Nikola Sprigg, Marilyn James, Marion F Walker, Kate Allatt, Rajnikant Mehta, Anand D Pandyan

**Affiliations:** 1 Faculty of Medicine and Health Sciences, University of Nottingham, Nottingham, UK; 2 Faculty of Health Sciences, University of Southampton, Southampton, UK; 3 Sheffield, UK; 4 Research Design Service East Midlands, University of Nottingham, Nottingham, UK; 5 School of Health and rehabilitation, Keele University, Keele, UK

**Keywords:** QUALITATIVE RESEARCH, PAIN MANAGEMENT, Occupational Therapy

## Abstract

**Introduction:**

Approximately 70% of patients with stroke experience impaired arm function, which is persistent and disabling for an estimated 40%. Loss of function reduces independence in daily activities and impacts on quality of life. Muscles in those who do not recover functional movement in the stroke affected arm are at risk of atrophy and contractures, which can be established as early as 6 weeks following stroke. Pain is also common. This study aims to evaluate the feasibility of a randomised controlled trial to test the efficacy and cost-effectiveness of delivering early intensive electrical stimulation (ES) to prevent post-stroke complications in the paretic upper limb.

**Methods and analysis:**

This is a feasibility randomised controlled trial (n=40) with embedded qualitative studies (patient/carer interviews and therapist focus groups) and feasibility economic evaluation. Patients will be recruited from the Stroke Unit at the Nottingham University Hospitals National Health Service (NHS) Trust within 72 h after stroke. Participants will be randomised to receive usual care or usual care and early ES to the wrist flexors and extensors for 30 min twice a day, 5 days a week for 3 months. The initial treatment(s) will be delivered by an occupational therapist or physiotherapist who will then train the patient and/or their nominated carer to self-manage subsequent treatments.

**Ethics and dissemination:**

This study has been granted ethical approval by the National Research Ethics Service, East Midlands Nottingham1 Research Ethics Committee (ref: 15/EM/0006). To our knowledge, this is the first study of its kind of the early application (within 72 h post-stroke) of ES to both the wrist extensors and wrist flexors of stroke survivors with upper limb impairment. The results will inform the design of a definitive randomised controlled trial. Dissemination will include 2 peer-reviewed journal publications and presentations at national conferences.

**Trial registration number:**

ISRCTN1648908; Pre-results. Clinicaltrials.gov ID: NCT02324634.

**Strengths and limitations of this study**

- This is the first study to target reciprocally the flexors and extensors, which although should have therapeutic benefits with no contraindications noted, has been ignored by previous electrical stimulation research and clinical practice.
- Explores the feasibility of recruiting patients and starting interventional treatment very early after stroke (within 72 h).
- Incorporates mixed methods and a feasibility economic component.
- Includes short-term and long-term follow-up (3, 6 and 12 months postrandomisation).
- The assessor is not blinded in this small-scale feasibility study, but methods for ensuring assessor blinding in the subsequent definitive trial will be explored.

## Introduction

Every year in England approximately 110 000 people have a stroke, making it the largest cause of adult disability.^1^ Hemiparesis is the single most disabling factor after stroke, affecting around 80% of patients.^2^ Approximately 70% of patients with stroke experience impaired arm function, which is persistent and disabling for an estimated 40%.^2^ Loss of function reduces independence in activities of daily living (ADL)^3^ and impacts on an individual's quality of life. Stroke survivors who do not recover functional movement in the paretic arm are more likely to have pain than those who recover arm function,^4^ and are at risk of muscle atrophy and contractures, which can become established as early as 6 weeks following stroke.^5^ There is some evidence that 60% of stroke survivors have contractures^6^ and they are most common in the upper limb.^7^

Recent audit data demonstrate that patients with stroke are unlikely to receive the recommended 45 min^8^ per day of each therapy required, for a minimum of 5 days per week.^3^ In the very acute stages of stroke rehabilitation, to enable discharge emphasis is often placed on regaining trunk control and mobility at the expense of upper limb rehabilitation. Average time spent in upper limb activities ranges from 0.9 to 7.9 min per therapy session.^9^

Several upper limb treatments are used in clinical practice, with varying success. Some treatments such as constraint-induced movement therapy and mirror box therapy have evidence of benefit^10–12^ but success is confined to patients with some active movement in the wrist and hand, and many patients do not. There is evidence to support early initiation of therapeutic interventions after stroke, and strong evidence that intensity of treatment is an important factor in recovery.^13–15^

Electrical stimulation (ES) therapy has potential value for those with reduced or no arm movement after stroke. ES delivers a small, harmless current that depolarises a nerve leading to two actions: a contraction of a muscle via a motor nerve; and a sensory signal to the brain and spinal cord by stimulation of the associated sensory nerves. Stimulation of the muscle causes a contraction similar to a naturally produced contraction which could prevent muscle atrophy. The sensory stimulation of the brain and spinal cord can contribute to neuroplasticity^16^ and reduction of pain.^4^ ^15^

Neuromuscular ES is a safe and harmless treatment for use in patient groups without the contraindications of uncontrolled epilepsy, pregnancy and those fitted with cardiac pacemakers. Metallic implants, cancerous lesions and risk of brittle bones around the stimulation site or the joint being treated are also contraindications. National clinical guidelines^2^ recommend routine use of ES for treating ‘foot drop’,^3^ but there is insufficient evidence to support the routine use in upper limb rehabilitation.^2^ Recently, Rosewilliam *et al*^17^ recruited 90 patients with no upper limb function 6 weeks after stroke and concluded that those who received ES of wrist extensors showed improved wrist extension, strength and grip. Secondary analysis concluded ES prevented the development of pain and deterioration in contractures.^4^

The ESCAPS study evaluates the feasibility of conducting a multicentre randomised controlled trial (RCT) of the efficacy and cost-effectiveness of early, intensive ES to prevent wrist joint deformities/contractures, weakness and upper limb pain after stroke. Qualitative components will explore barriers and facilitators to protocol adherence and clinical practice implementation.

The national clinical guidelines for stroke recommend that occupational therapists should aim to ‘re-educate’ the arm of patients who have reduced arm movement following stroke, by providing repetitive task training. This is inappropriate for many patients with stroke who do not have upper limb mobility. Treatment with ES does not require active movement; therefore, it could benefit patients who have severe to moderate arm function deficits.

This RCT is the first to study ES of flexors and extensors. Reciprocal stimulation of flexors and extensors will simulate the natural stretching movement and can be more effective in the prevention of contractures than stimulation of extensors alone. Flexor stimulation has been shown to reduce spasticity rather than being a contraindication.^18^ ^19^

In order to prevent deconditioning of the forearm muscles, there is a need to initiate treatment within 72 h of stroke. Premature discontinuation of therapy may reduce any therapeutic effect. No extended trials have been conducted, so it is important to establish compliance and attrition rates.

To ensure that the results from the definitive trial are generalisable, cost-effective and implementable, the research-related ES treatment will be delivered by National Health Service (NHS) therapists instead of specialist research therapists. The best strategy for self-management needs to be identified, so that treatment may continue at home after discharge.

This work will help form the basis for a definitive trial in health economics from a societal perspective. Loss of arm and hand function results in reduction of productivity, having a huge impact on individual patients and society.

The NHS needs to find effective new ways of managing the upper limb after stroke. This research is important because it addresses the needs of a significant proportion of patients with stroke. The ESCAPS feasibility study will inform the design of a definitive multicentre RCT to evaluate the efficacy and cost-effectiveness of early ES to the wrist flexors and extensors.

## Methods and analysis

### Study objectives

The primary objective of the study is to evaluate the feasibility of a RCT that will test the efficacy of delivering early, intensive ES to prevent poststroke complications (such as pain and contractures) to the paretic (weak) upper limb after stroke.

The objectives of this feasibility study therefore are to determine whether:

1. It is feasible to recruit within 72 h after stroke event, and to ascertain recruitment rates.
2. Initiating ES therapy within 72 h after stroke event is appropriate.
3. The ES regime proposed, that is, to start receiving ES on an acute stroke unit twice a day, 5 days a week and to continue with this schedule postdischarge for up to 3 months in total, is feasible, as measured by compliance, attrition and the qualitative work.
4. Study the interaction between compliance and response to treatment in order to identify the optimum compliance.
5. To determine preliminary health economic information surrounding the costs and benefits of the intervention.

We will also determine in this feasibility study what the most appropriate inclusion/exclusion criteria are; what the most sensitive and acceptable outcome measures are; the optimum method for single blinding and any barriers to compliance to the protocol from both the therapist and patient perspectives.

### Participants

The study is set in Nottingham, England. Participants will be patients recruited from the Stroke Unit of the Nottingham University Hospitals NHS Foundation Trust within 72 h of stroke event.

### Inclusion criteria

Inclusion:

- Confirmed clinical diagnosis of stroke;
- First stroke event to affect their upper limb;
- Aged >18 years;
- Impaired arm movement and strength caused by stroke (determined by the National Institute for Health Stroke Scale^20^ (NIHSS) arm subscore).

### Exclusion criteria

Exclusion:

- An existing chronic upper limb condition (eg, peripheral nerve injury);
- Cardiac pacemaker;
- Pregnancy;
- Epilepsy;
- Undiagnosed pain or skin conditions (not related to the stroke).

### Recruitment

For this feasibility study, we aim to recruit 40 participants over a period of 17 months from one centre (the Nottingham Stroke Unit at the Nottingham University Hospitals NHS Trust). The Clinical Research Network (CRN) stroke research officers will be notified by the clinical ward staff of newly admitted (within 72 h post-stroke) patients with stroke who meet the eligibility criteria. The clinical staff will have screened the patient against the eligibility criteria and those patients that meet the eligibility criteria will be informed of the study, and asked if they give voluntary consent. If the participant lacks capacity to consent for themselves, consent will be sought from an approved consultee according to the Mental Capacity Act. A stroke research officer will visit the patient on the ward to explain the study further and answer any questions.

### Study design

The ESCAPS study is a quantitative single-centre feasibility RCT (n=40) with an embedded qualitative component to evaluate patient recruitment within 72 h of stroke to receive either routine care with ES or routine care only.

The RCT is designed as a parallel group, two-arm superiority trial with 1:1 allocation ratio.

The embedded qualitative component will explore:

- Practical implementation issues such as acceptability of ES treatment to patients and adherence to ES treatment protocol,
- Potential barriers and facilitators to incorporation of ES treatment into routine practice, such as training of therapy staff.

Using:

1. Three focus groups with physiotherapists (n=4) and occupational therapists (n=4) involved in delivering and supporting the ES intervention;
2. Semistructured interviews with a purposive subsample representing a range of ages, gender and stroke severity of patient participants and their nominated carers (intervention group (n=10); control group (n=5)).

Preliminary economic analysis will test the feasibility of collecting economic data using a patient resource use questionnaire.

**Figure 1:**
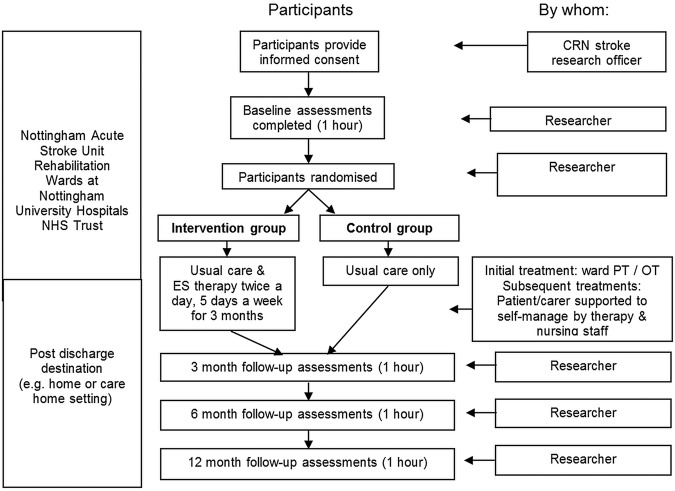
Schematic diagram of the schedule and time commitment for trial participants in each study group (CRN, Clinical Research Network; ES, electrical stimulation; NHS, National Health Service).

A schematic diagram of the schedule and time commitment for trial participants in each study group is included as figure 1.

### Randomisation

Following baseline measures, participants will be randomised to ES therapy intervention or usual care control. All participants eligible for inclusion will be randomised on a 1:1 basis. Concealed random allocation will be conducted by a statistician using a computer-generated pseudo-random list with random permuted blocks of varying sizes. Allocation concealment will be ensured by the statistician preparing the randomisation codes in sealed envelopes that will only be opened by the researcher after the patient has been recruited and has completed all baseline measurements. Participants will be stratified into four groups (n=10):

1. No arm movement (Action Research Arm Test (ARAT)^21^ score=0) and cognitive impairment (Montreal Cognitive Assessment (MoCA)^22^ <26);
2. No arm movement (ARAT^21^ score=0) but no cognitive impairment (MoCA^22^ >26);
3. Some arm movement (ARAT^21^ score >1) and cognitive impairment (MoCA^22^ <26);
4. Some arm movement (ARAT^21^ score >1) but no cognitive impairment (MoCA^22^ >26).

A minimisation technique will be applied during randomisation with minimisation on: age, sex, side of stroke and severity of arm weakness (as measured by the NIHSS^20^ arm subscore).

### Intervention treatment

Treatment with current controlled ES is delivered using a two channel constant current stimulation (maximum output 100 mA, pulse width 450 μs and a frequency between 40 and 60 Hz as per participant preference)^i^. The motor points for stimulation are selected to produce reciprocal flexion and extension through full range of movement.^23^ The initial assessment and treatment session with an NHS band 5 or above physiotherapist or occupational therapist will involve identification of the motor points for the forearm flexors and extensors, and placement of self-adhesive electrodes over these motor points. The current intensity will be increased to produce an alternating contraction of the flexors and extensors using a flex-hold-extend-hold pattern, ensuring that a pure movement is produced with no ulnar or radial deviation. The skin will be marked with a skin-safe marker pen to show the correct area to place the electrodes for future treatments and the ES device will be locked to the selected settings. A single stimulation and hold cycle will last 20 s and this will be cyclically repeated for 30 min after which the device can be removed.

Clinical staff will assist the patient to apply the electrode pads to the premarked motor points and switch on the device to the prestored treatment setting (this will take between 2 and 5 min) for subsequent treatments. The device will provide treatment without the need for a therapist to be present.

Prior to hospital discharge, the patient and/or nominated carer will be taught how to self-manage the treatment where possible. Treatment will continue twice a day, 5 days a week (Monday to Friday), for a total period of 3 months.

Modifications will not be made to the intervention. A participant may discontinue their allocated intervention or withdraw from the study for the following reasons: withdrawal of consent, or changes to their health status preventing their continued participation. Whenever possible, study participants will be retained in the trial to enable follow-up data collection and prevent missing data.

### Usual care control

Participants randomised to the control group will not receive ES therapy but will receive all usual care. ‘Usual care’ will include all the therapy interventions that are standard practice on the Nottingham Stroke Unit. As per national clinical guidelines,^2^ the application of ES to the upper limb is not standard practice. It is not possible to provide a placebo device without the possibility of it being easily identified as such. One reason is because movement is seen in the upper limb when the ES device is activated; furthermore, participants will be recruited and start treatment while on the hospital ward, so control and intervention participants may be in close proximity on the same wards as each other and would likely notice a difference between the intervention and placebo devices.

### Adherence to treatment

As part of the feasibility study data on adherence to the intervention treatment will be collected using two different methods. The ES devices have an internal memory function that will record data on usage. In addition, the study participants and/or their nominated carers will be asked to complete a patient diary to show the days and times that they have used the device. These data will be collected during the 3-month outcome assessment visit.

### Concomitant interventions during the trial

Participants enrolled on the ESCAPS trial cannot be enrolled in any other concomitant trials involving the application of experimental upper limb interventions that may impact trial outcomes.

### Blinding

It will be difficult to blind this study as the investigator and patient cannot be blinded during the procedure. However, an objective of this feasibility is to ascertain the most appropriate blinding procedure for the ensuing definitive trial.

The lead ward therapist will be informed of group allocation as they (or a nominated qualified therapist) will be providing the initial treatment. We will take every step to minimise allocation and outcome bias. Trial participants will not be blinded to group allocated because they will need to be informed as to whether they have been allocated to the intervention group receiving the ES therapy, or the control group who will not receive any ES therapy intervention.

The researcher responsible for the completion of outcome measures will not be blinded to group allocation as data on compliance will need to be collected via the use of participant diaries that will be collected at the 3-month follow-up assessment. This researcher will also have to record data on the number of ES treatments recorded (to measure home compliance) by the ES devices’ internal memory function (current intensity in mA and total time the device is used).

### Outcome measures

The primary outcomes relate to the feasibility outcomes of the study.

1. Feasibility of the trial design:
  - Recruitment rates: number/per cent of participants recruited within 72 h post-stroke; time post-stroke that participants received their first treatment;
  - Recruitment strategy: number/per cent of patients screened, number/per cent eligible and approached, number/per cent who consented, number/per cent excluded after screening;
  - Completion/attrition rates: number/per cent of participants who completed the intervention;
  - Compliance/adherence to treatment protocol: number/per cent of participants who received ES twice a day, 5 days a week while in hospital, and number/per cent who continued with the treatment regime after discharge. Mean, minimum and maximum number of ES treatments that participants received during the 3-month intervention period;
  - Consultee consent rates: number/per cent of patients unable to give informed consent, and number/per cent consented by a consultee, number of consultees who declined consent;
2. Tolerability: number of participants who withdraw or decline intervention; records of interventions declined and why;
3. Integrity of the study protocol: measured by examining how many participants are able to complete the study, number/per cent of missing data, and number/per cent of people who completed each of the outcome measures at 3-month, 6-month and 12-month follow-up, calculation of the cost of running the study; analysis of the qualitative interview and focus group data. The secondary outcomes relate to clinical outcome measures. Demographic characteristics (age, gender, ethnicity and socioeconomic status), stroke characteristics (date, type and side of stroke), cognitive status (MoCA^22^) and premorbid function state (Nottingham Extended ADL (NEADL)^24^) will be collected at baseline.

Participants will complete the following assessments at 0, 3, 6 and 12 months:

- Neurological outcome (NIHSS^20^ score);
- Independence in daily activities (Barthel ADL Index score^25^ and modified Rankin Scale (mRS)^26^ ^27^);
- Pain in the affected arm (Scale of Pain Intensity (SPIN)^28^);
- Muscle contractures/muscle activity (Biometrics EMG equipment);
- Arm function (ARAT^21^);
- Stroke-related quality of life (Stroke Specific Quality of Life Scale (SS-QOL))^29^;
- Health status (EuroQol-5D (EQ-5D)^30^);
- Patient resource use questionnaire;
- Carer strain (Caregiver Strain Index (CSI)^31^) will be completed by the participant's nominated carer.

### Data collection methods

Following recruitment to the trial, a stroke researcher will visit the participant on the ward to obtain the baseline measurements. In cases where it is not possible to obtain all the outcome measurement data from the patient or the medical records, for the BI, NEADL, mRS, resource use questionnaire, the researcher will obtain by proxy data from the next of kin. A stroke researcher will visit the participant at their place of discharge to complete the 3-month, 6-month and 12-month follow-up assessments.

### Sample size

This is a feasibility study and therefore the sample size is determined by the number of participants that is feasible to study within the timescale of the project. Consecutive sampling will be used to recruit 40 participants. This will provide sufficient information to indicate feasibility. Outcome data will be utilised to inform a sample size calculation for the definitive trial by assessing variability and completeness of data.

### Data management

Case report forms (CRFs) and all measurement data (with the exception of the spasticity and contracture measurement data) will be completed on paper forms. Data will then be entered and stored in a password protected electronic database. The original paper copies of CRFs and all study data will be treated as confidential documents and held securely in accordance with regulations. Participants will each be assigned a unique trial identity code for use on all study documents. All source documents will be filed at the principal investigator's site.

### Data analysis

Data will be statistically summarised/analysed using SPSS, and MathCad will be used to extract measures related to spasticity and contractures. Analyses will cover the following:

- Total patients recruited within 72 h of stroke event; average length of time post-stroke when patients received first treatment.
- Total patients screened, eligible and approached, consented, and excluded after screening.
- Total patients who completed the intervention; number who completed 3-month, 6-month and 12-month follow-up assessments.
- Total participants receiving ES per protocol (PP); mean, minimum and maximum number of ES treatments received during 3-month intervention period; qualitative patient/carer interview data, and ES machine's memory which records number of sessions and duration.
- Total patients unable to give informed consent; number consented by consultee; number of consultees who declined.
- Recruitment and attrition rates, number of patients lost to follow-up and reasons.
- Quality of health economic data between assessment points especially 6–12 months regarding completeness. Cross referencing of data against medical records where possible. Sensitivity of the EQ-5D in measuring outcome. Combined cost and outcome analysis to determine potential cost-effectiveness of ES verses usual care.
- Thematic analysis will be used to identify themes representative of patient experience of treatment, and the experience of carers with the application of the ES device.

As the sample size is small and a method of minimisation is used, we propose to estimate effect size (using the Cohen's d and the 95% CI) for the main study measures that are interval level data. The OR (and the 95% CI) will be estimated when data are dichotomous. When measures are ordinal, nominal relative risk ratios will be used to present all forms of adverse events.

In addition to estimating effect size, we will explore the use of the following statistical techniques to analyse our data: The group of patients randomised to receive the active treatment will be compared with the control group for all our primary outcomes variables recorded. Continuous variables that are normally distributed will be analysed using generalised linear modelling techniques. Where repeated measures have been recorded, we will apply generalised estimating equation techniques, considering appropriate correlation structure, with time interaction. For continuous variables, treatment differences will be presented with associated 95% CIs. For all statistical tests p<0.05 (2-sided) will be considered statistically significant.

No additional secondary analyses (eg, subgroup or adjusted analyses) are planned for this feasibility study.

For this study, the following definitions of analysis population will be followed:

*All-available-patient population*: All patients who have consented for the study will be used for patient accountability and listings and will include patients who are randomised but who do not receive their ES intervention treatment.

*Intention-to-treat (ITT) population*: All patients who have been randomised and received their ES treatment intervention at baseline and who complete follow-up assessment at 3 months, 6 months and 12 months will be included in an ITT population.

*The PP population*: All patients included in the ITT population, who participate in the study, without major protocol violation.

Missing values will be imputed by using the last value carried forward. We acknowledge that this method is associated with a risk of bias in patients who are deteriorating.

### Trial status

The official study start date was 1 April 2015. Recruitment to the feasibility trial began on the 1 June 2015 with the first patient entered into the study on 5 June 2015.

## Ethics and dissemination

### Informing potential trial participants of possible benefits and known risks

The stroke research officer will provide the patient and their consultee (if appropriate) with a verbal explanation of the trial and written patient information sheets, ensuring that the participant has sufficient time to consider participating or not. The CRN research officer will answer any questions that the participant has concerning study participation.

### Obtaining informed consent from participants

Trained stroke research officers will obtain written informed consent from patients willing to participate in the trial. The informed consent form will be signed and dated by the participant before they enter the trial and before they undergo any interventions (including history taking) related to the study. Patients who are unable to sign their name because the stroke has affected their dominant hand and they are unable to sign a legible signature with their non-dominant hand will be asked to point to a box within the consent form and a witness will sign to state that the patient wished to give informed consent to participate in the study.

An ‘aphasia friendly’ version of the information sheets and consent form will be available for those with communication difficulties. Such aphasia friendly information sheets and consent forms have been used in previous stroke trials by the study team.

Patients who lack the mental capacity to consent to participate in the study will be recruited after consultation with their next of kin or nominated carer. Consultee advice will be obtained from the patient's relative or close friend who would know the patient's wishes. Capacity is a situation-specific assessment and the CRN staff and clinical stroke team have had training in assessing capacity. Those participants who lack capacity will be considered for recruitment to the main feasibility trial only and not participation in the patient and carer interviews.

Irrespective of patients’ mental capacity, verbal or gestured agreement will be sought prior to ES treatment, and objections will be respected. If a participant becomes distressed by the ES treatment, it will be stopped.

Participants who lack capacity to provide informed consent will not be recruited to the patient and carer interview part of the study, but their nominated carer may opt to be interviewed about their experience of supporting someone without capacity to participate in the trial.

Owing to the early delivery of ES within 72 h poststroke, it may be that obtaining informed consent from the patient is not possible due to communication and/or cognitive difficulties. In these circumstances, we will obtain consultee consent. However, if during the intervention period the patient regains their communication and/or cognitive function, we will obtain informed consent from the patient at that time.

### Study withdrawal

It will be explained to the potential participant or their consultee that entry into the study is entirely voluntary and that treatment and care of the participant will not be affected by their decision. Participants or their consultee will be informed that they can withdraw from the study at any given point and that this will not affect their future care. Participants may be withdrawn from the trial either at their own request or at the discretion of the investigator. The participants will be made aware that this will not affect future care. Participants will be made aware (via the information sheet and consent form) that should they withdraw, the data collected to date cannot be erased and may still be used in the final analysis.

Participants may discontinue their allocated intervention or withdraw from the study for the following reasons:

- Withdrawal of consent,
- Changes to their health status preventing their continued participation.

Enrolled participants who withdraw and were not yet randomised will be replaced (though the withdrawn participant will keep their trial ID). Participants who withdraw after randomisation will not be replaced.

### Data protection and confidentiality

All trial staff and investigators will endeavour to protect the rights of the trial's participants to privacy and informed consent, and will adhere to the Data Protection Act 1998. The CRF will only collect the minimum required information for the purposes of the trial. CRFs will be held securely in a locked filing cabinet with access limited to the trial staff. Computer held data including the trial database will be held securely on a dedicated web server and will be encrypted and password protected. Participant confidentiality will be ensured by utilising identification code numbers. Individual participant medical information obtained as a result of this study are considered confidential and disclosure to third parties is prohibited.

Access to the CRFs shall be restricted to those personnel approved by the principal investigator and recorded on the Trial Delegation Log. The full data set will be restricted to the Trial Management Group, Independent Trial Steering Consultant and Sponsor. On completion of the study and following dissemination of the findings, secondary anonymised data will be available on request.

### Research governance and the conduct of the trial

The trial is being conducted in accordance with the ethical principles that have their origin in the Declaration of Helsinki 1996, the principles of Good Clinical Practice, and the Department of Health Research Governance Framework for Health and Social Care 2005.

### Dissemination of research findings

As this is a feasibility study, the main dissemination strategy will be after the ensuing trial. The findings from this study will be disseminated through study reports, through publications in academic peer reviewed journals, and conference presentations. The results will be communicated to the participants and involved stroke unit staff in the form of a letter thanking them for their involvement and support of the study, along with a lay summary of the findings and plans for future research.

### Clinical trials authorisation

Clinical trials authorisation is not required.

### Trial management and regulatory issues

This study is being hosted by Nottingham University Hospitals NHS Trust and coordinated by the University of Nottingham (the research sponsor). The principal investigator (JCF-S) will manage the day-to-day running of the study. The Trial Management Group will meet quarterly throughout the trial to discuss progress, including recruitment, withdrawals, treatment compliance, clinical issues and dissemination plans. As this is a feasibility study, the funder does not require a formal Trial Steering Committee or Data Monitoring Committee. However, an independent Trial steering Consultant will monitor and supervise the progress of the study towards its interim and overall objectives; concentrate of progress of the trial, adherence to the protocol and consideration of new information of relevance to the research question; provide advice on all appropriate aspects of the trial; and agree proposals for substantial protocol amendments. This independent monitor will audit the core trial processes and documents

Any potential modifications to the protocol will be discussed and agreed by the Trial Management Group, Trial Steering Consultant and the research Sponsor. Permission for a formal amendment to the protocol will then be sought from the Research Ethics Committee who granted the original ethical approval for the study. The local NHS Research and Development office will then be notified of any approved amendments.

Table 1 shows all items from the WHO trial registration data set.

**Table 1:** All items from the WHO trial registration data set

Data category	Information
Primary registry and trial identifying number	ISRCTN Registration ISRCTN16489086
Date of registration in primary registry	15/01/2015
Secondary identifying numbers	ClinicalTrials.gov NCT02324634
Source(s) of monetary or material support	Monetary support: Department of Health funding from the NIHR RfPB programme (PB-PG-1013-32034) Material support: NeuroTrac Rehab devices used for the intervention were donated by Verity Medical Ltd
Primary sponsor	University of Nottingham
Secondary sponsor(s)	Nottingham University Hospitals NHS Trust
Contact for public queries	JCF-S (principal investigator) Joanna.fletcher-smith@nottingham.ac.uk
Contact for scientific queries	JCF-S (principal investigator) Joanna.fletcher-smith@nottingham.ac.uk
Public title	Early Electrical Stimulation to prevent Complications to the Arm Post-Stroke—a feasibility study (ESCAPS)
Scientific title	Early electrical stimulation to the wrist extensors and wrist flexors to prevent the post-stroke complications of pain and contractures in the paretic arm—a feasibility study
Countries of recruitment	England, UK
Health condition(s) or problem(s) studied	Stroke: prevention of post-stroke complications (eg, pain and contractures) in the upper limb
Intervention(s)	ES applied to the wrist extensors and wrist flexors for 30 min, twice a day, 5 days a week, for 3 months The ES will be set to deliver a 450 μs pulse at a frequency of 40–60 Hz (as per patient convenience). The intensity of the current will be increased to produce an alternating contraction of the flexors and extensors using a flex-hold-extend-hold pattern. A single stimulation and hold cycle will last 20 s and this will be cyclically repeated for 30 min Control group will receive usual care only and will not receive any ES treatment
Key inclusion and exclusion criteria	Inclusion criteria: Confirmed clinical diagnosis of first disabling stroke event affecting the upper limb Aged >18 years Impaired arm movement and strength (NIHSS arm subscore >0) resulting in reduced function, caused specifically by the stroke Exclusion criteria: Previous history of stroke affecting their upper limb Peripheral nerve injury/existing orthopaedic condition affecting the upper limb; fixed contractures at the elbow, wrist or fingers; malignancy in the area of the ES electrode placement; or epilepsy Cardiac pacemaker or similar implanted device Pregnancy Undiagnosed pain or skin conditions (not related to the stroke)
Study type	Feasibility RCT (single centre) Interventional Allocation: randomised Masking: unblinded (for practical reasons during the feasibility trial but in any subsequent pilot and definitive trials the outcomes assessor will be blinded)
Date of first enrolment	June 2015
Target sample size	40
Recruitment status	Recruiting
Primary outcome(s)	Pertain to the feasibility aims of the study: Feasibility of the trial design (12 months)—recruitment rates: number/per cent of participants recruited within 72 h post-stroke; time post-stroke that participants received their first treatment; recruitment strategy: number/per cent of patients screened, number/per cent eligible and approached, number/per cent who consented, number/per cent excluded after screening; completion rates: number/per cent of participants who completed the intervention; number/per cent of participants who received ES twice a day, 5 days a week while in hospital, and number/per cent who continued with the treatment regime after discharge. Mean, minimum and maximum number of ES treatments that participants received during the 3-month intervention period; recruitment of patients lacking mental capacity to consent for themselves: consultee consent rates (number/per cent of patients unable to give informed consent, and number consented by a consultee, number of consultees who declined consent) Tolerability (12 months)—proportion of participants who withdraw or decline intervention; records of interventions declined and why Integrity of the study protocol (12 months)—measured by examining how many participants are able to complete the study, per cent of missing data, and per cent of people who completed each of the outcome measures at 3-month, 6-month and 12-month follow-up, calculation of the cost of running the study
Key secondary outcomes	NIHSS score (0, 3, 6, 12 months)—neurological outcome and degree of recovery Barthel ADL Index score and modified Rankin score (0, 3, 6, 12 months)—independence (functional ability) in basic daily activities SPIN (0, 3, 6, 12 months)—pain in the affected arm Muscle contractures (reduction in range of movement and spasticity; 0, 3, 6, 12 months)—muscle contractures will be monitored by measuring muscle activity during assessments using Biometrics equipment ARAT (0, 3, 6, 12 months)—arm function SS-QOL (0, 3, 6, 12 months)—stroke-related quality of life EQ-5D (0, 3, 6, 12 months)—health status Patient resource use (cost) questionnaire (0, 3, 6, 12 months)—a measure of resource use and health-related costs CSI (0, 3, 6, 12 months)—carer strain NEADL (0 months)—premorbid functional state MoCA (0 months)—cognitive status at baseline

ADL, activities of daily living; ARAT, Action Research Arm Test; CSI, Caregiver Strain Index; ES, electrical stimulation; EQ-5D, EuroQoL-5D; ISRCTN, International Standard Randomised Controlled Trial Number; MoCA, The Montreal Cognitive Assessment; NEADL, Nottingham Extended ADL; NHS, National Health Service; NIHR, National Institute for Health Research; NIHSS, National Institute for Health Stroke Scale; RCT, randomised controlled trial; RfPB, Research for Patient Benefit; SPIN, Scale of Pain Intensity; SS-QOL, Stroke Specific Quality of Life Scale.

### Adverse events

The occurrence of an adverse event as a result of participation is not expected and no adverse event data will be collected. The ES device itself is not under investigation and these devices have already passed extensive safety testing and are considered safe for use by patients with a range of conditions including stroke. Such devices are currently used in some areas of clinical practice on an ad hoc basis. The manufacturer's advice will be followed with regard to excluding patients who have a contraindication (eg, implanted cardiac pacemaker, epilepsy, pregnancy, malignancy in the area of the electrode placement).

## Footnotes

i The NeuroTrac Rehab Verity Medical Limited in compliance with the European Union Medical Device Directive MDD93/42/EEC under the supervision of SGS, notified body number 0120 CE mark status. Verity Medical Ltd is certified by SGS to the following quality standards: ISO 9001:2008, ISO13485:2003.
